# Cross-Bridge Group Ensembles Describing Cooperativity in Thermodynamically Consistent Way

**DOI:** 10.1371/journal.pone.0137438

**Published:** 2015-09-11

**Authors:** Mari Kalda, Pearu Peterson, Marko Vendelin

**Affiliations:** Laboratory of Systems Biology, Institute of Cybernetics at Tallinn University of Technology, Akadeemia tee 21, 12618 Tallinn, Estonia; University of Minnesota, UNITED STATES

## Abstract

The aim of this work is to incorporate cooperativity into Huxley-type cross-bridge model in thermodynamically consistent way. While the Huxley-type models assume that cross-bridges act independently from each other, we take into account that each cross-bridge is influenced by its neighbors and cooperativity is induced by tropomyosin movement. For that, we introduce ensembles of cross-bridge groups connected by elastic tropomyosin. By taking into account that the mechanical displacement of tropomyosin induces free energy change of the cross-bridge group ensemble, we develop the formalism for thermodynamically consistent description of the cooperativity in muscle contraction. An example model was composed to test the approach. The model parameters were found by optimization from the linear relation between oxygen consumption and stress-strain area as well as experimentally measured stress dynamics of rat trabecula. We have found a good agreement between the optimized model solution and experimental data. Simulations also showed that it is possible to study cooperativity with the approach developed in this work.

## Introduction

In the heart, the mechanical work is tightly linked to energy conversion processes to ensure that the main function of the heart—pumping blood—is always possible. As a manifestation of a tight link between mechanics and energetics in the heart, it has been shown that oxygen consumption (VO_2_) of the heart is linearly related to pressure-volume area (PVA) [1]. Pressure-volume area is a specific area in pressure-volume diagram surrounded by end-systolic PV line, the end-diastolic line and the systolic segment of the PV trajectory for heart contraction. As a analog of PVA-VO_2_ relationship on tissue level, stress-strain area (SSA)-VO_2_ linear relationship has been demonstrated [2] and can be used for estimation of regional VO_2_ in the heart [3]. Earlier, we have shown that it is possible to reproduce PVA-VO_2_ relationship by the finite element model of the left ventricle [4] if the active properties of myocardium are described by the model that reproduces SSA-VO_2_ relationship [5].

As a part of regulatory mechanisms involved in the heart function, cooperative length-dependent activation of actomyosin interaction by calcium has been shown to play a major role in mechanical response of the heart [6]. While numerous mathematical models of heart contraction have been developed, accurate description of the cooperativity turned out to be problematic [7]. Among the developed approaches to model mechanical contraction, the models based on Huxley formalism or molecular dynamics simulations stand out by ability to link development of mechanical force to biochemistry in thermodynamically consistent manner [8, 9]. As a result, it is possible to simulate changes in mechanical force induced by changes in ATP, ADP, and Pi concentrations [10]. When compared with the molecular dynamics based approaches, deterministic nature of Huxley-type models makes them attractive for incorporation into mathematical models of the whole heart. However, while providing a strong theoretical base for linking mechanics and chemistry, Huxley-type models have been lacking description of cooperativity that would be thermodynamically consistent [5, 11]. For example, our earlier models while describing actin and myosin interaction in thermodynamically consistent manner, had a phenomenological description of Ca^2+^ activation to describe the sarcomere length dependency of the contraction [5, 12].

The aim of this work is to incorporate cooperativity of Ca^2+^ activation of actomyosin interaction into Huxley-type cross-bridge models in thermodynamically consistent way. Here, we give a description of theoretical framework of the developed approach that incorporates direct interactions between neighboring cross-bridges. Next, we demonstrate simulations performed by Huxley-type model using thermodynamically consistent description of the cooperative Ca^2+^ activation.

## Theory

### Huxley-type cross-bridge model

**Fig 1:**
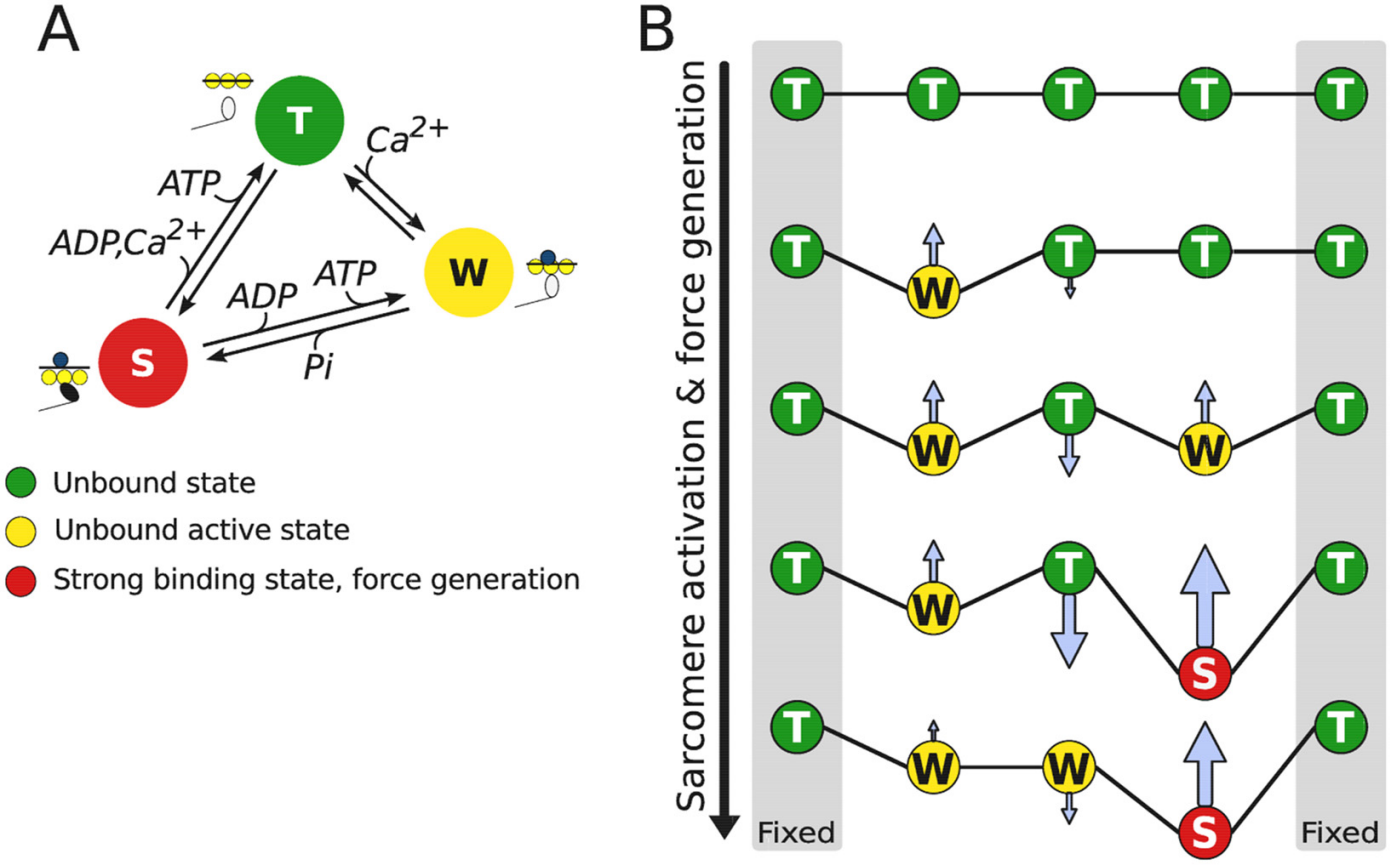
Computational model of the cross-bridge interaction. (A) Kinetic scheme of actin and myosin interaction used in three-state Huxley-type cross-bridge model. (B) Incorporating cooperativity into half-sarcomere activation and force generation. We consider an ensemble of five consecutive cross-bridges (binding states), out of which the first and the last ones are always in unbound state as boundary conditions. Arrows indicate the influences the neighboring binding states due to elastic deformation on tropomyosin, that connects all cross-bridges in a group, on binding of *Ca* ^2+^ (transition from T to W) or force generation (transition from W to S).

Before introduction of treatment of cooperativity, we give a description of the classical Huxley-type cross-bridge model. For simplicity, let as assume that actomyosin interaction can be described by three different discrete biochemical states, as in Fig 1A. This simplification is used only in the theoretical description. The considered states are as follows. In state T (unbound state), the myosin binding site on actin is “blocked” by tropomyosin. In state W (unbound active state), Ca^2+^ is bound to troponin-C and the binding site is “open” for myosin head to bind to actin. Finally, in state S (strong binding state), myosin head is strongly bound to the actin. The state S is only state where myosin head is able to generate force. As in [8], a cross-bridge is defined as a myosin head projection to binding sites on actin characterized by the above mentioned states.

In Huxley-type single-site model [8, 9], the force produced by attached cross-bridge is assumed to be elastic and it depends on the axial position *x* of the nearest actin site, relative to model-defined origin. For example, the origin could correspond to the position at which cross-bridge does not produce force in one of the force-producing states. Since cross-bridge and the nearest actin site have one-to-one correspondence, for brevity, the position of the nearest actin site will be referred as cross-bridge position in the following text. If *d* is the length of one regulatory unit (RU—one troponin-tropomyosin complex and the seven associated actins [13]) of thin filaments, then *x* is defined in the range between −*d*/2 and *d*/2; *d* ≈ 36nm [9]. According to T. Hill [8], the force *F* produced by the cross-bridge at position *x* is related to the free energy *G* of a particular state: *F* = ∂*G*/∂*x*. Linear dependency of force on *x* leads to quadratic dependence for free energy, an assumption that we use throughout of this paper. Regarding this model, as there is no force associated with the states T and W (*F*_T_ = *F*_W_ = 0), the corresponding free energy functions are constant with respect to *x*.

Such relationship between mechanical force and free energy provides a unifying link between chemical reactions and mechanics. Namely, the ratio of the reaction rate constants is determined by the difference in free energies. For first order transitions between states, say, *A* and *B*, described by forward and reverse rate constants *k*_*A*,*B*_ and *k*_*B*,*A*_, respectively, the ratio is as follows:

$$\begin{array}{c} \frac{k_{A,B}\left(x\right)}{k_{B,A}\left(x\right)}=\exp(-\frac{G_{B}\left(x\right)-G_{A}\left(x\right)}{RT}), \end{array}$$ (1)

where *R* and *T* stand for universal gas constant and absolute temperature, respectively. For the considered model in Fig 1A, *A* is either T, W, or S. For values of *x*, where *G*_S_ < *G*_W_ (model in Fig 1A), the strongly bound state is thermodynamically favorable; otherwise the unattached state is more favorable.

To describe the contraction of the muscle, we use kinetic formalism developed by T. Hill [8]. According to Hill’s formalism, cross-bridges can be divided into ensembles (called subensembles in [8]) parameterized by the position *x*: cross-bridges are in the same ensemble if their positions are within the range *x* and *x*+*dx*. For the fixed *dx*, the number of cross-bridges in ensembles is assumed to be the same and constant for any *x* due to the lack of register between myosin and actin. In a given ensemble (labeled by *x*), we define *n*_*A*_(*x*, *t*) as a fraction of those cross-bridges that at time *t* are in a state *A*. We have:

$$\begin{array}{c} n_{T}\left(x,t\right)+n_{W}\left(x,t\right)+n_{S}\left(x,t\right)=1. \end{array}$$ (2)

Changes in cross-bridge states are induced either by chemical transition from one state to another or by sliding of actin and myosin filaments relative to each other with the rate *v* = *v*(*t*) of half-sarcomere lengthening. For example, for state T, this would result in the following governing equation for *n*_T_(*x*;*t*):

$$\begin{array}{ccc} \frac{\partial n_{T}}{\partial t}+\frac{\partial n_{T}}{\partial x}v & = & k_{S,T}n_{S}+k_{W,T}n_{W}-(k_{T,S}+k_{T,W})n_{T}, \end{array}$$ (3)

where *k*_*A*,*B*_ = *k*_*A*,*B*_(*x*) are the first order kinetic rate constants for transition from state *A* to state *B*. Similar equations are found for *n*_W_(*x*, *t*) and *n*_S_(*x*, *t*).

The integral properties of the muscle, such as developed stress and ATPase rate are found from integration over ensembles [8]. For instance, the Cauchy stress *σ*_*a*_ developed by cross-bridges in a half-sarcomere is an integral force of all cross-bridge states in all ensembles [14]:

$$\begin{array}{c} \sigma_{a}\left(t\right)=\frac{ml}{d}\int_{-\frac{d}{2}}^{\frac{d}{2}}(n_{T}\left(x,t\right)F_{T}\left(x\right)+n_{W}\left(x,t\right)F_{W}\left(x\right)+n_{S}\left(x,t\right)F_{S}\left(x\right))\;dx, \end{array}$$ (4)

where the *m* is the number of cross-bridges in the unit volume and *l* is the length of the half-sarcomere. Taking into account the force relations of the current model, *F*_T_ = *F*_W_ = 0 and $F_{\text{S}}(x)=K_{\text{S}}(x-x_{\text{S}}^{\text{eq}})$, we have

$$\begin{array}{c} \sigma_{a}\left(t\right)=\frac{mlK_{S}}{d}\int_{-\frac{d}{2}}^{\frac{d}{2}}n_{S}\left(x,t\right)\left(x-x_{S}^{\text{eq}}\right)\;dx, \end{array}$$ (5)

where *K*_S_ and $x_{\text{S}}^{\text{eq}}$ are force constant and cross-bridge equilibrium position, respectively, corresponding to force generating state S.

The average cross-bridge ATP consumption rate is

$$\begin{array}{c} V_{\text{ATP}}\left(t\right)=\frac{1}{d}\int_{-\frac{d}{2}}^{\frac{d}{2}}(k_{S,W}\left(x\right)n_{S}\left(x,t\right)+k_{S,T}\left(x\right)n_{S}\left(x,t\right)\\ -k_{W,S}\left(x\right)n_{W}\left(x,t\right)-k_{T,S}\left(x\right)n_{T}\left(x,t\right))\;dx \end{array}$$ (6)

leading to the total ATP consumption per cross-bridge during a beat

$$\begin{array}{c} V_{\text{ATP}}^{\text{beat}}=\int_{0}^{t_{b}}V_{\text{ATP}}\left(t\right)\;dt, \end{array}$$ (7)

where *t*_*b*_ is the time period of one beat.

Note that while the thermodynamic considerations limit the ratio of rate constants Eq (1), the choice of one of the rate constant is not limited by the given equilibrium relationship. Thus, *k*_*A*,*B*_ can depend on position *x*, half-sarcomere length *l*, time *t*, and some other parameters as long as *k*_*A*,*B*_/*k*_*B*,*A*_ ratio satisfies Eq (1).

### Cooperativity in Huxley-type model

*General principles.* For treatment of cooperativity in the model, we assume that the cooperativity is induced by tropomyosin movement. When Ca^2+^ binds to troponin-C, tropomyosin will shift to expose a binding site on actin [15]. Binding of myosin to the exposed binding site will shift the tropomyosin even further [16]. Since tropomyosin is a spiral protein wrapped around actin helix, movement of tropomyosin influences its movement in the neighboring RU binding sites as well. Assuming that tropomyosin is an elastic string, the movement of tropomyosin induced by binding of Ca^2+^ or myosin head requires mechanical work. The amount of required mechanical work depends on the states of the neighboring RU binding sites on actin as these states carry also the displacement information of tropomyosin at these binding sites. In essence, our treatment of cooperativity involves formulation of free energy functions for a set of neighboring cross-bridges that includes energy required to move tropomyosin from its initial state to a new state taking into account the displacements of tropomyosin at neighboring RUs.

For simplicity, we assume that the cross-bridges are connected by an elastic string representing tropomyosin (Fig 1B). Attachment of Ca^2+^ to troponin-C (transition from state T to W) or attachment of myosin head to binding site on actin (transition from state W to S) deforms the string altering its stress state. As a result of tropomyosin deformation, depending on the state of neighbor cross-bridges, attachment (or any other transition of states) can be either less or more thermodynamically favorable due to the elastic forces induced by deformation of tropomyosin before and after attachment.

For illustration, let us consider the transitions of a short sequence of cross-bridges connected with tropomyosin as shown in Fig 1B. During attachment of Ca^2+^ by a troponin-C (transition from the top to the second row), tropomyosin is elongated between the first and the third cross-bridge (from the left). Such elongation requires additional mechanical work to be performed on the system leading to the increase of tropomyosin free energy after attachment. Same applies to the consecutive attachment of Ca^2+^ and transition to the strong binding state by one of cross-bridges (the third and forth rows from the top). However, attachment of Ca^2+^ to the central troponin-C is simplified due to the forces induced by tropomyosin deformation (transition to the last row). Thus, as it is shown in the example, changes in the cross-bridge states can be either hindered or facilitated due to the elastic deformation of tropomyosin induced by the neighbor cross-bridges.

To introduce cooperativity into Huxley-type model in thermodynamically consistent way, we assume that the muscle contraction can be described by ensembles of cross-bridge groups. In the classical Huxley-type models, cross-bridges are grouped into ensembles according to the position of the nearest actin binding site, as described in previous subsection. Cooperativity between cross-bridges is taken into account by generalizing the definition of ensembles by including the influence (state) of neighboring cross-bridges to a particular ensemble of cross-bridges. For that, let us assume that the states of *q* subsequent cross-bridges are related, that is, the transition of the state of one of these cross-bridges depends on the states of other cross-bridges. Ideally, *q* should be equal to the number of cross-bridges that are influenced by the same tropomyosin. This would ensure one-to-one correspondence between the mathematical model and the muscle mechanics. In practice, however, such a model would be difficult to realize because of its enormous size. Let us define a ensemble of cross-bridge groups consisting of *q* cross-bridges such that the positions of cross-bridges, (*x*_1_, *x*_2_, …, *x*_*q*_), are in a range between (*x*_1_, …, *x*_*q*_) and (*x*_1_ + *dx*_1_, …, *x*_*q*_ + *dx*_*q*_) for all members of the ensemble. We denote the states of cross-bridges in a group by *A* = (*α*_1_, …, *α*_*q*_), where *α*_*i*_ corresponds to the states of individual cross-bridges. We denote the set of individual cross-bridge states by 𝕊. In our example 𝕊 = {T,W,S}. To describe the state of cross-bridge group ensembles, we introduce the ensemble state density function *N*_*A*_(*x*_1_, …, *x*_*q*_;*t*), such that

$$\begin{array}{c} N_{A}\left(x_{1},\cdots ,x_{q};t\right)\;dx_{1}\cdots dx_{q} \end{array}$$ (8)

gives the fraction of ensembles at time *t* in group state *A* and its cross-bridge positions in a range between (*x*_1_, …, *x*_*q*_) and (*x*_1_ + *dx*_1_, …, *x*_*q*_ + *dx*_*q*_) among all possible ensemble configurations in terms of group states and cross-bridge position ranges:

$$\begin{array}{c} \sum_{A}\int_{-\frac{d}{2}}^{\frac{d}{2}}\cdots \int_{-\frac{d}{2}}^{\frac{d}{2}}N_{A}\left(x_{1},\cdots ,x_{q};t\right)\;dx_{1}\cdots dx_{q}=1, \end{array}$$ (9)

where summation is taken over all possible states of cross-bridges in a group, that is *A* ∈ 𝕊^*q*^.

Because the cooperativity between cross-bridges is determined by the movement of the tropomyosin and *q* is going to be much smaller than the number of cross-bridges that are influenced by the same tropomyosin, we need to define also the states of cross-bridges that are immediate left and right neighbors of the specified *q* cross-bridges. In this work we assume that the corresponding boundary cross-bridges are in unbound inactive state and tropomyosin is in the initial relaxed state, i.e. the boundary cross-bridge is always in state T. In Fig 1B, the example corresponds to ensembles formed by *q* = 3 cross-bridges with the additional boundary cross-bridges highlighted by gray areas. Although, the requirement of the boundary conditions ruins the one-to-one correspondence property of the model and the view of muscle, we presume that the cooperativity effects can be noticed within the possible artifacts introduced by these boundary conditions.

As for cross-bridges in the Huxley-type models, transitions between states of cross-bridge groups are driven by chemical reactions and the corresponding free energy profiles. It is assumed that all transformations in the cross-bridge group happen as separate reactions at different time moments for different cross-bridges within a group. Thus, as elementary processes of the considered system, chemical reactions involve only one cross-bridge or troponin-C. For example, only one Ca^2+^ attachment can take place at some time moment, not two calcium molecules binding simultaneously to the group.

The kinetics and force generation of the cross-bridge group is driven by the free energy change. Free energy *G*_*A*_ of the group in state *A* is defined as a sum of free energies of all cross-bridges (*G*_*α*_*i*__) in the group and free energy of tropomyosin influencing them (*U*_*A*_):

$$\begin{array}{c} G_{A}\left(x_{1},\cdots ,x_{q}\right)=U_{A}+\sum_{i=1}^{q}G_{\alpha_{i}}\left(x_{i}\right). \end{array}$$ (10)

In our formulation, we assume that tropomyosin is connected to actin at the locations of troponin complexes with the same spatial period *d* as RUs have. For example, when Ca^2+^ binds, the tropomyosin moves only at the corresponding connection point with the resulting elastic deformation of tropomyosin accommodating to the new configuration. In general, the location of myosin head is stochastic process, so is also subsequent tropomyosin deformation. For simplicity, we assume that the change of tropomyosin free energy in transition from W to S does not depend on the binding location of myosin head. With this assumption, we can compute the free energy of tropomyosin (*U*_*A*_) as if the myosin head always binds at the location of tropomyosin connection points. As a result, free energy of tropomyosin depends only on cross-bridge group state *A* and not on position of each of the cross-bridges (*x*_1_, …, *x*_*q*_). This is a manifestation of *U*_*A*_ not depending on (*x*_1_, …, *x*_*q*_) in Eq (10). The free energy of tropomyosin, with the group of cross-bridges in state *A*, is a sum of free energies of all tropomyosin fragments in the group:

$$\begin{array}{c} U_{A}=U_{T;\alpha_{1}}+U_{\alpha_{1};\alpha_{2}}+\ldots +U_{\alpha_{q};T}, \end{array}$$ (11)

where *U*_*α*;*β*_ denotes the free energy of a tropomyosin string fragment between two neighboring cross-bridges being in respective states *α* and *β*. Thus, we have to define the free energies of all possible fragments to determine the system. An example of the free energy formulation is given in the Methods section as a part of the description of implemented model.

The shortening or lengthening of the half-sarcomere would lead to the same displacement of the cross-bridges in the group from the closest actin binding sites. This important property of the system has to be taken into account when considering permissive changes of cross-bridge groups within the *q*-dimensional space. As a result, and taking into account Eq (10), the mechanical force produced by the group of cross-bridges is the sum of the forces produced by cross-bridges in the group:

$$\begin{array}{c} F_{A}\left(x_{1},\cdots ,x_{q}\right)=\frac{\partial G_{A}\left(x_{1}+\xi ,\cdots ,x_{q}+\xi \right)}{\partial \xi }{|}_{\xi =0}=\sum_{i=1}^{q}\frac{\partial G_{\alpha_{i}}\left(x_{i}\right)}{\partial x_{i}}=\sum_{i=1}^{q}F_{\alpha_{i}}\left(x_{i}\right), \end{array}$$ (12)

where partial derivative of *G*_*A*_ is taken with the respect of the permissive changes in the *q*-dimensional space.

Taking into account that the cross-bridges groups can undergo transitions induced either by chemical reaction or changes in half-sarcomere length, the kinetics of cross-bridge cycling is described by the following system of partial differential equations for ensemble state density function *N*_*A*_(*x*_1_, …, *x*_*q*_, *t*):

$$\begin{array}{c} \frac{\partial N_{A}}{\partial t}+\frac{\partial N_{A}\left(x_{1}+\xi ,\cdots ,x_{q}+\xi ,t\right)}{\partial \xi }{|}_{\xi =0}v=\sum_{B}(k_{B,A}N_{B}-k_{A,B}N_{A}), \end{array}$$ (13)

where *k*_*A*,*B*_ = *k*_*A*,*B*_(*x*_1_, …, *x*_*q*_, *t*) is the rate constant for transition of the group from state *A* to *B*, and *v* = *v*(*t*) is the rate of the half-sarcomere lengthening.

Taking into account that only one cross-bridge can perform a transition at any given time, as specified earlier in the definition of considered elementary processes, *k*_*A*,*B*_ is non-zero only for such pair of group states *A* and *B* where only one cross-bridge is changed (for example, transition from state TTT to TWT): *B* = (*α*_1_, …, *β*_*j*_, …, *α*_*q*_) where *A* = (*α*_1_, …, *α*_*j*_, …, *α*_*q*_) and *j* is the index of cross-bridge undergoing a state change. As before,

$$\begin{array}{c} \frac{k_{A,B}\left(x_{1},\ldots ,x_{q},t\right)}{k_{B,A}\left(x_{1},\ldots ,x_{q},t\right)}=\exp(-\frac{G_{B}\left(x_{1},\ldots ,x_{q}\right)-G_{A}\left(x_{1},\ldots ,x_{q}\right)}{RT}). \end{array}$$ (14)

Taking into account the considered elementary processes and partitioning of the free energy of the group Eqs (10) and (11), it is easy to show that the free energy difference between states *A* and *B* depends only on one cross-bridge position and not on positions of other cross-bridges in the group. In particular,

$$\begin{array}{c} G_{B}-G_{A}=G_{\beta_{j}}\left(x_{j}\right)-G_{\alpha_{j}}\left(x_{j}\right)+U_{\alpha_{j-1},\beta_{j}}+U_{\beta_{j},\alpha_{j+1}}-U_{\alpha_{j-1},\alpha_{j}}-U_{\alpha_{j},\alpha_{j+1}} \end{array}$$ (15)

where *x*_*j*_ is the position of the cross-bridge involved in the reaction.

From *N*_*A*_, the mechanical stress produced by a half-sarcomere can be found similar to the classical Huxley-type models Eq (4). For Cauchy stress *σ*_*a*_, the following integral has to be found:

$$\begin{array}{c} \sigma_{a}\left(t\right)=ml\int_{-\frac{d}{2}}^{\frac{d}{2}}\cdots \int_{-\frac{d}{2}}^{\frac{d}{2}}\sum_{A}N_{A}\left(x_{1},\cdots ,x_{q},t\right)\;F_{A}\left(x_{1},\cdots ,x_{q}\right)\;dx_{1}\cdots dx_{q}. \end{array}$$ (16)

To solve the system Eq (13) for *N*_*A*_, we introduce distribution function of cross-bridge groups *γ* and the fraction of cross-bridge groups *n*_*A*_ in state A among groups forming ensemble at (*x*_1_, …, *x*_*q*_):

$$\begin{array}{c} \gamma \left(x_{1},\ldots ,x_{q},t\right)=\sum_{A}N_{A}\left(x_{1},\ldots ,x_{q},t\right), \end{array}$$ (17)

$$\begin{array}{c} N_{A}\left(x_{1},\ldots ,x_{q},t\right)=\gamma \left(x_{1},\ldots ,x_{q},t\right)n_{A}\left(x_{1},\ldots ,x_{q},t\right), \end{array}$$ (18)

$$\begin{array}{c} \sum_{A}n_{A}\left(x_{1},\ldots ,x_{q},t\right)=1. \end{array}$$ (19)

As shown in Appendix (Aim 1), the distribution function *γ* has the following general form:

$$\begin{array}{c} \gamma \left(x_{1},\ldots ,x_{q},t\right)=\gamma_{0}\left(x_{1}-a\left(t\right),\ldots ,x_{q}-a\left(t\right)\right), \end{array}$$ (20)

$$\begin{array}{c} a\left(t\right)=\int_{t_{0}}^{t}v\left(\tau \right)d\tau , \end{array}$$ (21)

where *γ*_0_ is an initial distribution of the groups at *t* = *t*_0_. Thus, cross-bridge group position in *q*-dimensional space is altered only through the changes in half-sarcomere length.

In addition, as shown in Appendix (Aim 2), *n*_*A*_ obeys

$$\begin{array}{c} \frac{\partial n_{A}^{\prime }}{\partial t}+\frac{\partial n_{A}^{\prime }}{\partial x_{1}^{\prime }}v=\sum_{B}\left(k_{B,A}^{\prime }n_{B}^{\prime }-k_{A,B}^{\prime }n_{A}^{\prime }\right), \end{array}$$ (22)

after coordinate transformation

$$\begin{array}{c} x_{1}^{\prime }=\frac{1}{q}\sum_{i=1}^{q}x_{i}, \end{array}$$ (23)

$$\begin{array}{c} x_{i}^{\prime }=x_{i}-x_{1},i=2,\ldots ,q, \end{array}$$ (24)

with $n_{A}^{\prime }$ and $k_{A,B}^{\prime }$ representing *n*_*A*_ and *k*_*A*,*B*_ in a new coordinate system $\left(x_{1}^{\prime },\ldots ,x_{q}^{\prime }\right)$. Notice that $n_{A}^{\prime }$ dynamics can be solved by integrating system of partial differential equations in *t* and $x_{1}^{\prime }$ while $x_{2}^{\prime },\ldots ,x_{q}^{\prime }$ are parameters.

By selecting different *γ*_0_, different assumptions regarding cross-bridge groups can be tested. For example, in a classical Huxley-type model, the lack of register between myosin and actin leads to constant *γ*_0_.

**Fig 2:**
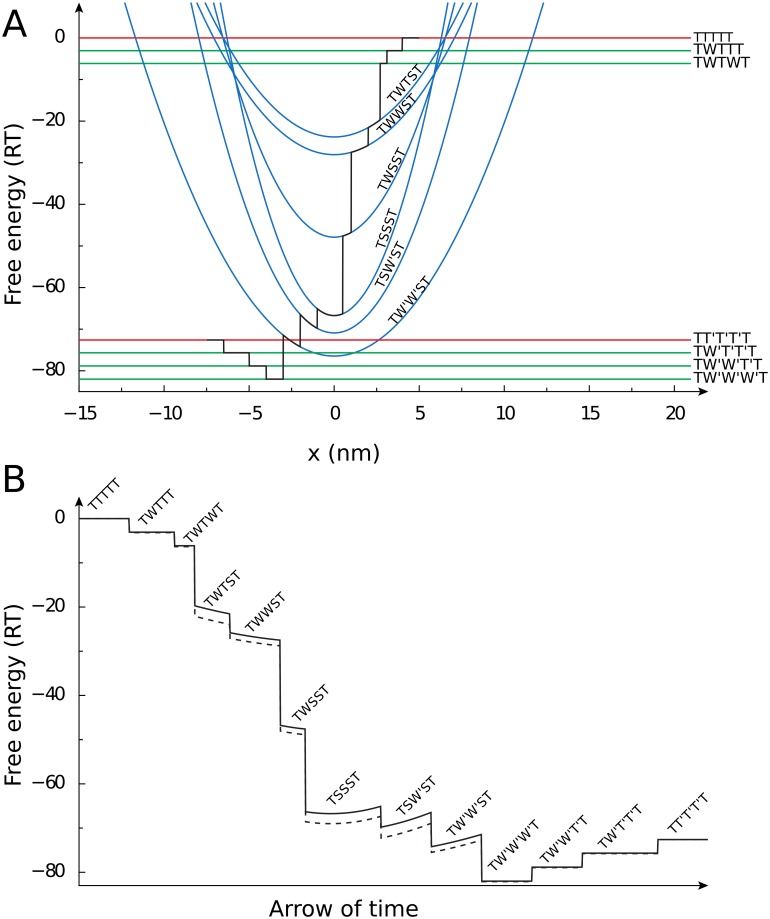
Scheme of free energy profiles and influence of cooperativity. (A) Scheme of one possible set of free energy profiles and transition trajectory (black solid line) from state where all five considered cross-bridges are in the unbound state (TTTTT) to state where three active cross-bridges are in unbound state with three ATP molecules hydrolyzed in reaction (TT’T’T’T). (B) Illustration of tropomyosin deformation influence on free energy of the cross-bridge group ensemble. The change of the free energy during reaction is shown for the simulation that takes into account the tropomyosin deformation (solid line) and for the simulation that does not take it into account (dashed line). Note that when tropomyosin deformation is considered, the change in free energy of the ensemble induced by one of the cross-bridges depend on the states of other cross-bridges in group.

*Example.* To illustrate how cooperativity would influence free energy profile of the group, let us consider the example in Fig 2A. In this example, cross-bridge group with *q* = 3 performs a series of transformations from unbound state TTT to producing mechanical force in state SSS and returning to state TTT. During this process, three ATP molecules have been hydrolyzed to ADP and Pi. To distinguish the state where the cross-bridge has hydrolyzed ATP, an’ symbol has been used next to the state (T’ and W’). Let us follow the hypothetical transition trajectory as shown in black in Fig 2A. In the beginning of the process (states TTT, WTT, and WTW), Ca^2+^ binds to two troponin-C’s in the group, but do not produce any force. As a result, the free energy of the group is independent of cross-bridge position. On the transition to state WTS, one of the cross-bridges produce mechanical force leading to the parabolic relationship between the free energy and cross-bridge position. Further binding of Ca^2+^ to the second troponin-C in the group (state WWS), shifts the free energy downwards, but does not change the steepness of the relationship. However, formation of the second and the third strong-binding cross-bridge state (group in states WSS and SSS) leads to the change in the shape of free energy dependency on cross-bridge position due to the larger force produced by the group. The process is reversed by subsequent unbinding of the cross-bridges and Ca^2+^. When we follow the changes in free energy of the group in time along this hypothetical trajectory, we can illustrate the role of tropomyosin deformation in free energy of the group (Fig 2B). Note how addition of tropomyosin deformation component changes the free energy profile (solid line) if compared with the free energy profile of the reaction without tropomyosin deformation (dashed). Several reactions are made either more or less thermodynamically favorable, as it is evident from the changes in steps induced in the free energy during transitions from one state to another.

*Simplifications used in the implemented model.* There are several simplifications to the general theory presented above that were introduced while implementing the model. To limit the size of the model, as well as to test its influence we considered the cases where *q* was 1, 3, or 4.

The next set of simplifications reduces the dimensionality of the model. Namely, if each of the cross-bridges can be in any of *K* states (*K* = 3 for example in Fig 1 with the state being either T, W, or S) then the system of partial differential equations describing cross-bridge kinetics Eq (13) consists of *K* ^*q*^ equations. Those equations have to be solved in *q*-dimensional space to find evolution of *N*_*A*_(*x*_1_, …, *x*_*q*_, *t*) in time. While the solution can be obtained by partitioning equations into a system of 1+1-dimensional partial differential equations with *q* − 1 parameters, as in Eq (22), the solution of these equations requires extensive computational time. Additionally, as model parameters, multi-dimensional rate constants *k*_*A*,*B*_(*x*_1_, …, *x*_*q*_, *t*) have to be specified. While *k*_*A*,*B*_ are restricted by the free energy difference between states *A* and *B* Eq (15), this still requires specification of large number of multi-dimensional functions as a model parameters. In practice, such formulation leads to very large computational requirements.

To study the effects of cooperativity induced by tropomyosin displacements, we made several simplifications in the implemented model. First, it is assumed that the rate constants *k*_*A*,*B*_(*x*_1_, …, *x*_*q*_, *t*) depend only on one *x*_*i*_ that corresponds to *i*-th cross-bridge in the group ongoing the change during the reaction. For example, the rate constant for transition from state TTT to TWT can be written as

$$\begin{array}{c} k_{\text{TTT},\text{TWT}}\left(x_{1},x_{2},x_{3},t\right)=k_{\text{TTT},\text{TWT}}\left(x_{2},t\right). \end{array}$$ (25)

Second, we assumed that all cross-bridges positions in the group are equal *x*_*i*_ = *x*_0_. Note that this assumption reduces the dimensionality of the system Eq (13) and corresponds to the following choice of *γ*_0_:

$$\begin{array}{c} \gamma_{0}\left(x_{1},\ldots ,x_{q}\right)=\frac{1}{d}\;\delta \left(x_{2}-x_{1}\right)\cdots \delta \left(x_{q}-x_{1}\right), \end{array}$$ (26)

with *δ* denoting Dirac delta function. As shown in Appendix (Aim 2), the model equations are then

$$\begin{array}{c} \frac{\partial n_{A}\left(x,t\right)}{\partial t}+\frac{\partial n_{A}\left(x,t\right)}{\partial x}v\left(t\right)=\sum_{B}(k_{B,A}\left(x,t\right)n_{B}\left(x,t\right)-k_{A,B}\left(x,t\right)n_{A}\left(x,t\right)), \end{array}$$ (27)

$$\begin{array}{c} \sigma_{a}\left(t\right)=\frac{ml}{d}\int_{-\frac{d}{2}}^{\frac{d}{2}}\sum_{A}n_{A}\left(x,t\right)\;F_{A}\left(x\right)\;dx. \end{array}$$ (28)

## Results

The following simulations were performed using a five-state model, that detailed description is given in Methods section. When compared with the three-state model (Fig 1), the five-state model has three force-producing states instead of one. This allows to describe the movement of myosin head by shifting free-energy minima between two force-producing states and detachment of calcium from troponin-C while the cross-bridge is in force-producing state.

The aim of these simulations is to demonstrate that the model can be used in practice to reproduce different dynamic aspects of heart muscle contraction. Here, we fitted the model solutions to (i) reproduce stress developed during isometric contraction at different sarcomere lengths, (ii) relationship between ATP consumption and stress-strain area during isometric and physiologic contractions, and (iii) relationship between end-systolic sarcomere length and stress in isometric and physiologic contractions.

We compared the optimal solutions obtained by the model with and without cooperativity. The model with cooperativity had three cross-bridges in the group (*q* = 3), positioned between unattached cross-bridges, as shown in Fig 1B. For the model without cooperativity, same model equations were used with *q* = 1 and tropomyosin free energy changes induced by cross-bridge cycling and calcium attachments were set to zero. As a result, that model neglected influence of cooperativity and was equivalent to the model without cooperativity, i.e. classical Huxley-type model formulation.

**Fig 3:**
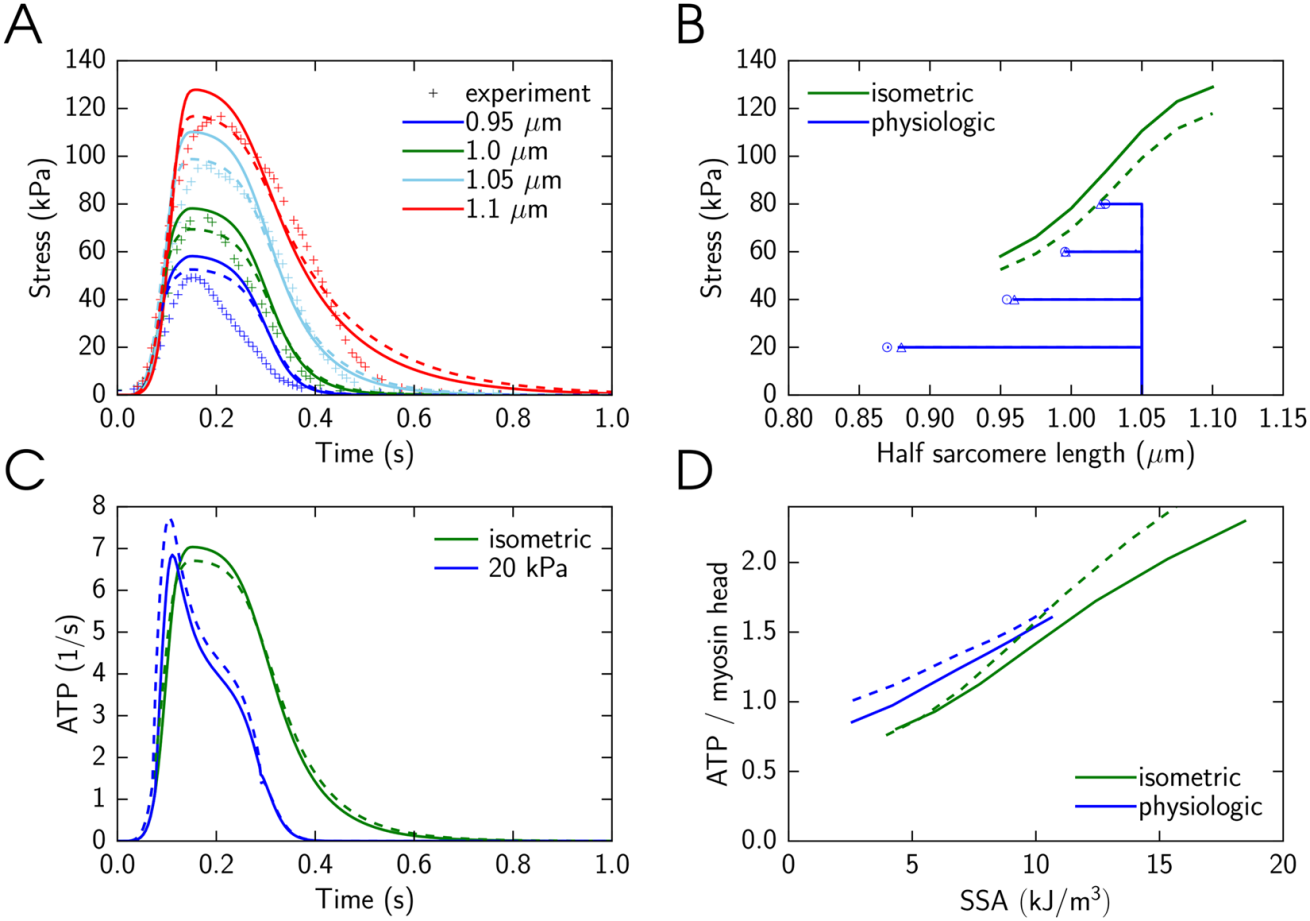
Use of the model with cooperativity between cross-bridges to simulate dynamic properties of the heart muscle contraction. Here, the solutions obtained with three interacting cross-bridges (*q* = 3, solid line) or a single cross-bridge (*q* = 1, dashed line) are shown. In these simulations, the model parameters were found by fitting model solution against the experimental data. A: Isometric contraction as a function of time at different half-sarcomere lengths from 0.95 *μm* to 1.1 *μm* compared with experimental measurements [20]. Sarcomere length is encoded in color, as indicated in the inset. B: End-systolic relationship between sarcomere length and stress for the isometric contractions are shown. In addition, changes in sarcomere length and developed stress are shown for physiologic contraction with the end-systolic value indicated by triangle (*q* = 3) or circle (*q* = 1) at different afterloads from 20 kPa to 80 kPa. C: ATP consumption by a cross-bridge during isometric and physiological contractions (20 kPa). Note how the difference in afterload changes ATP consumption by a cross-bridge. Here, simulations are shown for half-sarcomere end-diastolic length of 1.05 *μ*m. D: Total amount of consumed ATP molecules per myosin head during a cardiac cycle as a function of SSA for isometric and physiologic contractions. Note that the both models reproduce the linear relationship between SSA and energy consumption.

**Fig 4:**
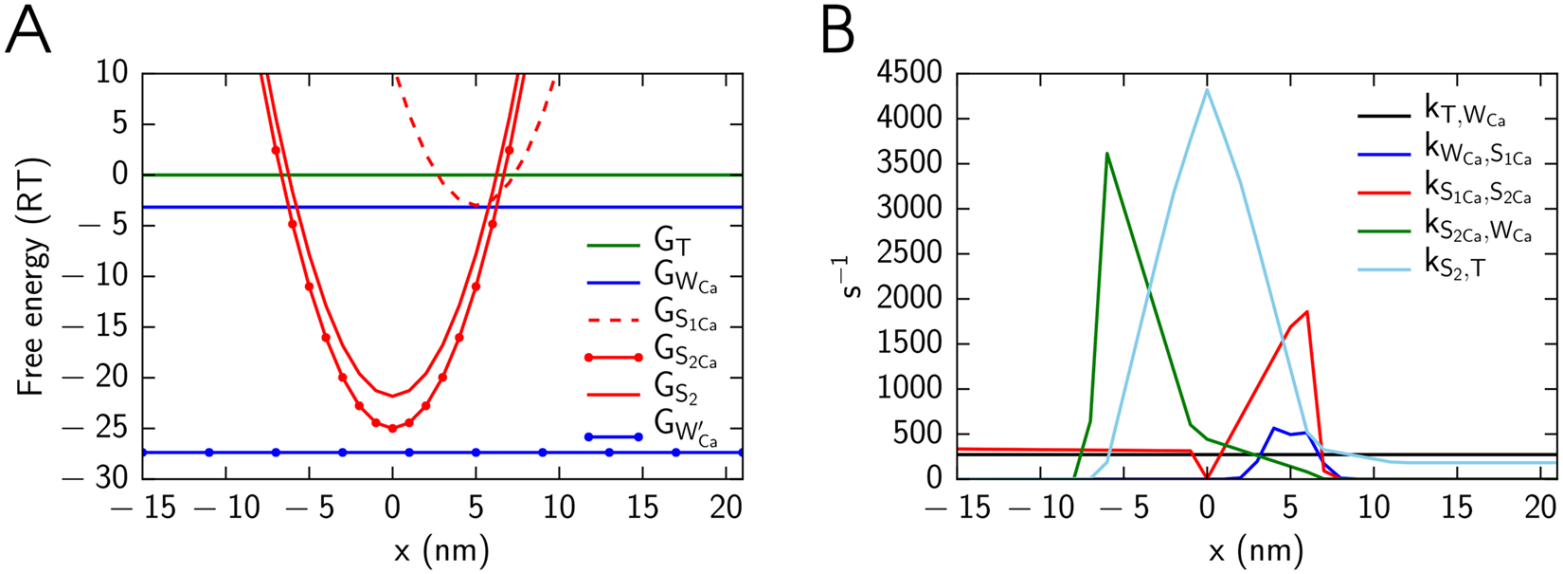
Free energy profiles (A) and cross-bridge forward rate constants (B). The model parameters were found by optimization procedure for the model with cooperativity induced by tropomyosin deformation (*q* = 3).

The optimized simulation results for the model with and without cooperativity are shown in Fig 3. During the optimization, the model parameters were varied to fit model solution against measured developed stress in isometric contraction (Fig 3A), to obtain the same end-systolic relationship for isometric and physiologic contractions (Fig 3B), and to obtain the same and linear relationship between ATP consumption and stress-strain area (Fig 3C) for isometric and physiological contractions. The forward rate constants found by fitting for the case with cooperativity are shown in Fig 4B. As described in Methods, tropomyosin free energy is given in the model through free energies of two tropomyosin segment configurations. The values of the free energies found by fitting of the model solution were *U*_T;W_ = 0.1RT and *U*_W;S_ = 0.05RT.

The both models—with and without cooperativity—were able to fit the data reasonably well. The main difference between the models is in the better reproduction of maximal stress dependency on sarcomere length during isometric contractions by the model with cooperativity (*q* = 3) than by the model without cooperativity (*q* = 1). We have also observed that the isometric relaxation phase is faster when cooperative interaction between cross-bridges is considered (Fig 3A). However, it could be due to the differences in the model parameter values, such as rate constants, and cannot be solely attributed to the effects of cooperativity.

When simulating the end-systolic relationship between developed stress and half-sarcomere length, the both models had similar end-systolic relationships for isometric and physiologic contractions. As it is clear from Fig 3B, in these simulations, the model without cooperativity (*q* = 1) obtained the end-systolic relationships that were marginally closer to each other in isometric and physiologic cases, when compared to the model with cooperativity (*q* = 3).

We calculated the amount of ATP molecules hydrolyzed during a contraction by each myosin head and related it to stress-strain area (SSA). For that, ATP consumption by a cross-bridge was found (Fig 3C) and integrated over time. The simulations were performed at different afterloads (physiological contractions) and different sarcomere lengths (isometric contractions) to obtain ATP consumption corresponding to different SSA values. In accordance with the experimental data [2], the calculated relationship between ATP consumed in a beat and SSA is linear and the same for shortening and isometric contractions (Fig 3D). The contraction efficiency, as determined by SSA and ATP consumption calculated by the model, was 74.4% and 61.0% for model with and without cooperativity, respectively. To estimate the efficiency, the model solution with the maximal SSA was used and we took into account the myosin ATPase concentration [17] of 0.18 mM (0.18 mol m^−3^) and free energy change during ATP hydrolysis of 60 kJ mol^−1^ [1, 18]. The simulation results are in good agreement with experimental data. Namely, it was found that chemomechanical efficiency of cross-bridge cycling is in the range of 60-70% [1].

To find out whether the differences between model solutions in Fig 3 are caused by the different model parameters found by the optimization or cooperativity effect, we performed the simulations with the same model parameters for the models with the different number of active cross-bridges in a group: *q* = 1, *q* = 3, and *q* = 4. For comparison, we used the set of parameters found by optimization for the model with cooperativity, *q* = 3 (Fig 3). Note that in this case, the parameters were found with the non-zero tropomyosin free energies *U*_W;T_ and *U*_W;S_. Use of these free energies lead to modifications in the free energy profile, as illustrated in Fig 2. For comparison purposes, we used the same tropomyosin free energies for the model with *q* = 1. Namely, in this case, a single cross-bridge that is able to change its state was located between two fixed cross-bridges. As a result, such case corresponds to non-cooperative model, but it takes into account some changes in free energy profile induced by the deformation of tropomyosin. Note that when we used the same model parameters but with zero tropomyosin free energies, the model solution was significantly disturbed that we attribute to the incompatibility between the rate constants and changes in free energy profile (results not shown).

**Fig 5:**
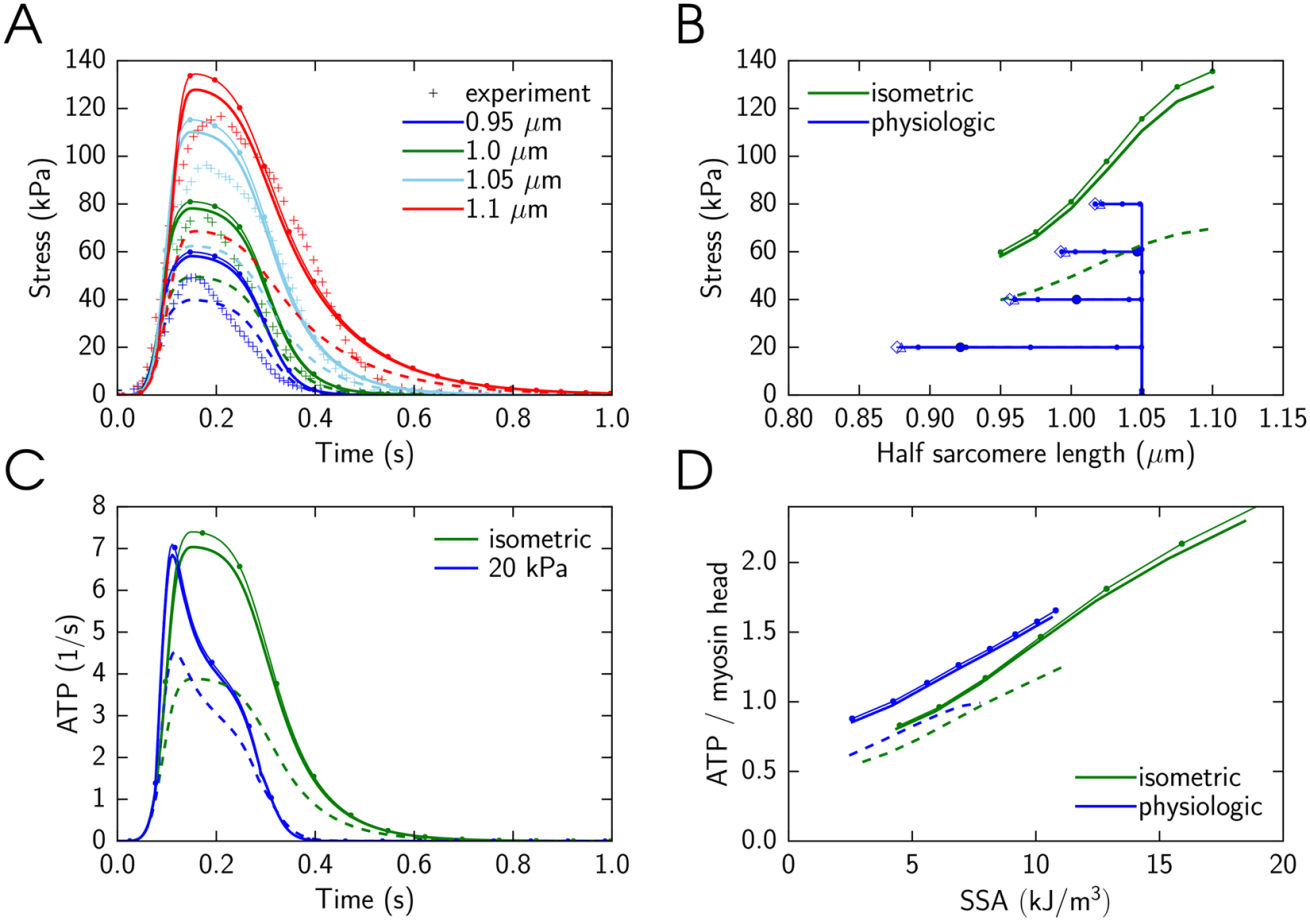
Influence of cross-bridge interaction on force generation and contraction. Here, the number of interacting cross-bridges was varied from one cross-bridge between the fixed ones (*q* = 1), to three and four (*q* = 3 or 4, respectively) active cross-bridges. The simulations were performed with the same model parameters, found by fitting for the model with *q* = 3. The subplots are as in Fig 3, with the addition of one more solution (*q* = 4) marked by line with dots and the end-systolic value indicated by diamond. Due to lack of influence from the neighboring binding state to free energy profiles, developed stress at the case where *q* = 1 is significantly lower than other two cases. Simulation results for *q* = 3 and *q* = 4 are similar and sometimes overlapping in the figure.

As shown in Fig 5, the results are different for different modes of cooperativity. The models with cooperativity (*q* = 3 and *q* = 4) give the solutions that are very similar to each other. In contrast, the model without cooperativity (*q* = 1), the peak isometric force is smaller (Fig 5A) and sarcomere length has different dynamics during physiologic contractions (Fig 5B). The contraction efficiency was also different for models with and without cooperativity (Fig 5D). Thus, this example clearly demonstrates strong effects of interaction between cross-bridges in the same group on model solution indicating that this approach can be used to study cooperativity of muscle contraction.

**Fig 6:**
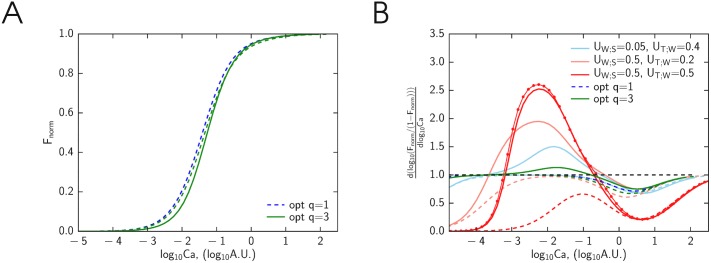
Calcium binding cooperativity measures calculated by the models with the different number of interacting cross-bridges. Here, the steady state solution of models is shown depending on calcium concentration with concentration given in arbitrary units at half-sarcomere length of 1.05 *μ*m. The model solutions are designated by colors with green and blue depending on whether the parameters were found by fitting the data using *q* = 3 or *q* = 1 model, respectively; and by dashed, solid and line with dots, depending on whether the simulations were done using *q* = 1, *q* = 3 or *q* = 4 model, respectively. A: Normalized force-calcium relationship found by the model. All solutions demonstrate the low cooperativity indicating that this aspect of the model has to be refined. B: The Hill coefficient calculated from the slope of force-calcium relationship in log-log scale. For non-cooperative binding, the slope is expected to be 1 (black dashed line). In addition to the solutions found for optimized model parameters, we calculated the Hill coefficient for model solutions with the larger free energy changes induced by tropomyosin deformation (the free energies of the segments are given in the figure legend). Note how the increase in tropomyosin free energy changes induced by cross-bridges and calcium binding leads to the increase of the Hill coefficient in the models with cooperativity effects included (*q* = 3 and 4) in contrast to the model with only one cross-bridge that can change its state (*q* = 1).

To demonstrate that the model with multiple cross-bridges can be used to study cooperativity in Ca^2+^ binding, we calculated the force produced by the cross-bridges for different Ca^2+^ concentrations. In these simulations, Ca^2+^ concentration in the cell was taken constant and the steady-state solution was found (Fig 6). According to our simulations, the cooperativity of calcium binding is rather low in all models with the optimized parameters (Fig 6A). To check the cooperativity at different levels of calcium, we calculated the slope from the Hill’s plot (taking derivative from log(*F*_*norm*_/(1 − *F*_*norm*_))—log[Ca^2+^] relationship, Fig 6B). As clearly demonstrated in Fig 6B, the Hill’s coefficient was about 1 for models with optimized model parameters. Note that during our optimization we did not use experimental force-calcium relationships. To see whether the larger Hill coefficients can be observed, we analyzed solutions with the larger interactions between cross-bridges (larger *U*_W;T_ and *U*_W;S_ values). Some of these solutions are shown for the model with *q* = 3 (solid line) or *q* = 4 (solid line with bullets) in Fig 6B. Note how the increase in changes in free energy of tropomyosin induced by its stretching leads to increase in Hill’s coefficient. Intriguingly, when the same model parameters were used for the model with *q* = 1 (only one cross-bridge between two fixed cross-bridges, dashed line), the Hill coefficient was always smaller or equal to one (Fig 6B). In addition, we observed that the Hill coefficient was consistently higher at the lower range of Ca^2+^ concentration (smaller than the concentration required to develop half of the maximal force) than on the higher Ca^2+^ concentrations. This is consistent with the experimental results of Dobesh et al [19] where the similar asymmetry of cooperativity in Ca^2+^ binding was reported. Thus, the simulation results in (Fig 6) demonstrate that cooperativity can be studied by this approach. However, as all our simulations with calcium binding in steady-state demonstrate, the model has to be developed further by either incorporating more interacting cross-bridges (increasing *q*) or variation of kinetic constants to reproduce high cooperativity of calcium binding observed in experiments.

## Discussion

The main result of this work is the development of thermodynamically consistent approach to incorporate cooperativity between cross-bridges into Huxley-type models. By extending the notation of ensembles to consecutive cross-bridges influenced by the same tropomyosin and taking into account that mechanical deformation of tropomyosin requires mechanical work, we were able to derive equations describing the dynamics of cross-bridge interaction. The equations are based on T.L. Hill formalism and take into account microscopic reversibility. In terms of Razumova et al [13] nomenclature, the developed formalism describes RU-RU, XB-XB, and XB-RU classes of cooperativity, where XB and RU refer to cross-bridge and troponin-tropomyosin regulatory unit, respectively. To illustrate the use of derived formalism, an example model has been constructed and we demonstrate that it is possible to apply such model to study force generation and ATP consumption by heart muscle.

The experiments used in our model simulations were based on the same selection as our earlier simulations [5]. Namely, the model was tested against the data on isometric twitch and ATPase properties of the heart muscle. While multiple models are able to reproduce isometric force generation and many other aspects of mechanical contraction of the heart muscle [21][11][22], the link between ATP consumption by cross-bridges and force generation has been difficult to reproduce [23, 24]. According to large body of experimental evidence, at the same inotropic state, oxygen consumption of heart is linearly related to pressure-volume area [1]. On tissue level, the same relationship holds when oxygen consumption is related to stress-strain area [2]. In our earlier models, we were able to reproduce the relationships on tissue and left ventricular levels [4, 5]. However, in these models, the activation of cross-bridges was driven by phenomenological description of troponin-C which included cooperativity effects. While cross-bridge cycling was described in thermodynamically consistent manner taking into account microscopic reversibility, the dynamics of troponin-C activation was given as a function of sarcomere length without detailed kinetic mechanisms involved in calcium activation of the muscle. Such phenomenological description was sufficient for demonstrating that relationship between pressure-volume area and oxygen consumption of the muscle can be reproduced using Huxley-type models and highlighted the importance of twitch duration prolongation with the increase of sarcomere length to reproduce this property [5]. In this work, we seek for a thermodynamically consistent formalism of cooperativity effects that would allow to address the mechanistic aspects of cooperativity in future studies.

Many different approaches, with the varying levels of complexity, have been applied to study cooperativity in the heart muscle contraction [7]. Many models approach cooperativity using phenomenological descriptions [22]. While these models are advantageous to study contraction phenomena, there are always concerns on applicability of the models in the corner cases that were not considered during the model design and parameters estimation. To introduce mechanistic aspects of cooperativity, several spatial models have been constructed which describe muscle contraction using Monte Carlo or Ising approaches [7, 25, 26]. While in many models the description of cooperativity includes variation of forward or reverse rate of one or several reactions depending on the state of the neighboring cross-bridges breaking the microscopic reversibility, several models stand out by tracking the changes in kinetic constants induced by neighbor interactions and ensuring microscopic reversibility of reactions [25, 27]. However, these models have been used only on steady-state conditions to study cooperative calcium binding. In this work, we describe the model that ensures microscopic reversibility of the reactions similar to [25, 27] by taking into account that deformation of tropomyosin requires investment of chemical free energy. By extending the notation of ensembles to group of neighboring cross-bridges, we were able to overcome the difficulties of introducing cooperativity in thermodynamically consistent manner into Huxley-type models [11].

Our approach to introducing cooperativity into the Huxley-type model is similar to the approaches used by Zou and Phillips [28] as well as Campbell et al [29]. In these models, the free energy variations induced by the movement of tropomyosin were taken into account through their modification of the corresponding rate constants in cross-bridge kinetics. As in our study, the state of the neighbor cross-bridges was taken into account when finding the changes in deformation of the tropomyosin induced by the transition of the cross-bridge or troponin-C from one state to another [28, 29]. While there is a similarity in thermodynamically consistent description of cooperativity, there are also clear differences between our approach and the earlier models [28, 29]. Namely, by using Huxley-type model and T.L.Hill approach linking the development of mechanical force with the free energy of the cross-bridge [8], the model ensures that mechanical work performed by the muscle is strictly consistent with the energy available to the cross-bridge through ATP hydrolysis. This is in contrast to the models that assume a fixed mechanical force developed by the cross-bridge in force-producing state that is not related to the deformation of the cross-bridge, as in [28, 29]. Through theoretical framework developed in our study, it is possible to model the mechanical contraction of the heart muscle, from cooperative binding of Ca^2+^ and cross-bridges to the force development, in thermodynamically consistent manner.

There are several simplifications that were introduced in formulation of the model. As one of the main simplifications in the model, we assumed that we can describe the position between equilibrium position of myosin heads and closest binding sites using one variable. Here, we assumed for simplicity, that the cross-bridge position is equal for all cross-bridges in the same group. In sarcomere, the same tropomyosin connects actin binding sites that would interact with different myofilaments. As a result, there is no trivial relationship between cross-bridges binding to consecutive actin binding sites and the system of partial differential equations Eq (13) has to be solved for *q*-dimensional fractions *N*_*A*_. Notice that the system of PDEs can be parameterized using certain coordinate transformation (see Eqs (22)–(24) leading to parameterized system of (1+1)-dimensional PDEs that can be numerically integrated in parallel, and hence, solving the full system Eq (13) is feasible. However, for illustration of the Huxley-type model extension presented in this work, we avoided the parameterization by fixing the cross-bridge positions within one cross-bridge group relative to each other. As common in the field, we used the single binding site assumption. Namely, cross-bridge is assumed to be able to attach only to one site on actin, as in [8]. Multi-site attachment is possible by extending the kinetic schemes of actomyosin interaction as in [30]. However, as opposed to Huxley-type cross-bridge models, we include interaction between cross-bridges leading to the notation of ensembles that involve a group of cross-bridges. The developed formalism can be used for more complicated cases than shown in this work. For example, it is possible to apply the same approach to the case where movement of tropomyosin next to one cross-bridge induces modification along the tropomyosin influencing all cross-bridges in the group.

In our simulations, we fitted only limited dataset of experiments (Fig 3). As clearly shown in Fig 6, the model with the found parameter values does not reproduce cooperativity of Ca^2+^ binding. While higher level of cooperativity has been demonstrated for model solutions with some other parameter sets, further optimization or model modification is needed to reproduce these data by the model.

As an application for the developed formalism and the mathematical models based on it, we envision the use of Huxley-type models for description of muscle active properties in simulations of the heart mechanics. As we have demonstrated earlier [4], it is possible to incorporate Huxley-type model into finite element model of the left ventricle. The numerical formulation of the finite element models of the heart requires finding elastic deformation of the heart wall and, if the active stress is described using the models that depend on rate of sarcomere shortening, finding the rate of deformation. Finding elastic deformation and the rate of deformation of the heart wall requires solution of a system of non-linear equations with deformation and the rate of the different parts of the wall influencing each other. As a part of iterative process usually used to find heart wall deformation, the developed active stress has to be calculated when given the deformation and its rate as an input. While use of Huxley-type models would make this part of calculations condiderably larger than a use of a small system of ordinary differential equations to describe active stress generation by sarcomeres, it is possible to run this part of calculations in parallel by calculating the active stress for each of the finite element nodes separately. With the increase of computational capacities available for researcher, usually in form of larger computer clusters, we think that the use of thermodynamically consistent Huxley-type models can help to study the cases where chemical environment in the cell, such as concentrations of ATP, ADP, inorganic phosphate and pH, is significantly different from control conditions.

In summary, we present extension of Huxley-type models that describes cooperativity of cross-bridge dynamics in thermodynamically consistent manner. The developed formalism demonstrates that it is possible to use deterministic models such as the model described in this work to study cooperativity of the muscle contraction.

## Methods

### Model description

**Fig 7:**
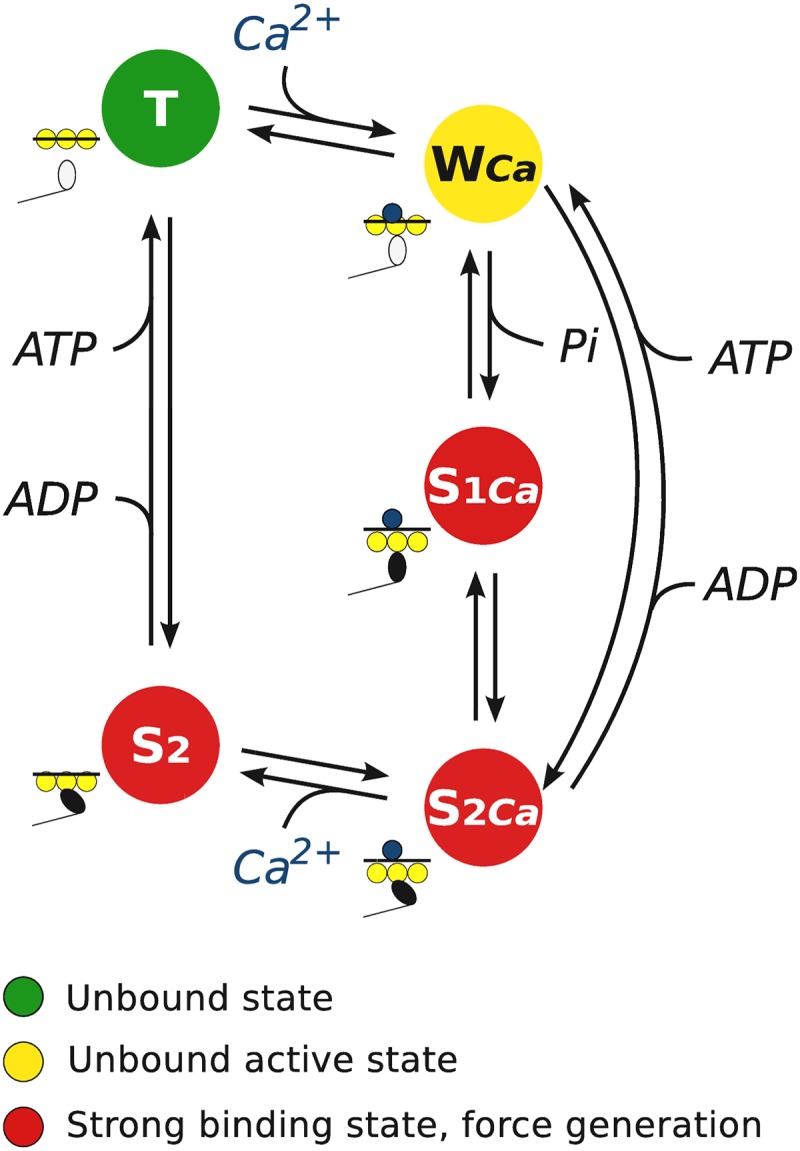
Scheme of five-state cross-bridge model. There are two biochemical states where no force is generated: unbound state T, unbound active state W_Ca_, where Ca^2+^ is bound to troponin-C. The strong binding state S is splited into three biochemical states where force is generated: S_1Ca_, S_2Ca_, where Ca^2+^ is bound to troponin-C and S_2_, where Ca^2+^ is unbound.

*Cross-bridge states.* In our simulations we consider five-state cross-bridge mathematical model (𝕊 = {T,W_Ca_,S_1Ca_,S_2Ca_,S_2_}) with the kinetic scheme for each of the cross-bridges shown in Fig 7. In this mathematical model we have two biochemical states where no force is generated (T and W_Ca_) and three force-generating states (S_1Ca_, S_2Ca_, and S_2_). Out of these states, W_Ca_, S_1Ca_, and S_2Ca_ have Ca^2+^ bound to associated troponin-C.

*Tropomyosin displacement and associated free energy changes.* As described in theory, cooperativity of cross-bridges is introduced by taking into account that the binding of calcium or cross-bridge leads to a displacement of tropomyosin. Since tropomyosin connects all cross-bridges in a group, the elastic deformation of tropomyosin will influence the free energy of the group as well as reaction kinetics. Assuming linear relationship between elastic force and deformation, the elastic energy of tropomyosin fragment in between neighboring cross-bridge sites that are in state *A* and *B*, respectively, is

$$\begin{array}{c} E_{A;B}=U_{tr}+\int_{d}^{l_{A;B}}\left(\xi -d\right)\;{𝓝}_{A}\;K_{tr}d\xi =U_{tr}+\frac{1}{2}\;{𝓝}_{A}\;K_{tr}\;{\left(l_{A;B}-d\right)}^{2}, \end{array}$$ (29)

where *l*_*A*;*B*_ is the length of tropomyosin fragment, *d* is minimal possible length of the fragment that is equal to the distance between neighboring actin binding sites (36 nm), *K*_*tr*_ is stiffness of single tropomyosin (21.6 pN/nm, [31]), 𝓝_*A*_ is Avogadro constant, and *U*_*tr*_ is elastic energy of relaxed tropomyosin:

$$\begin{array}{c} U_{tr}=\frac{{𝓝}_{A}K_{tr}d^{2}}{2}. \end{array}$$ (30)

At 37C, *U*_*tr*_ ∼ 3400RT. In the considered five-state cross-bridge model, there are three different positions where tropomyosin can shift, we denote those positions as T, W, S. We have

$$\begin{array}{ccc} E_{T;W} & = & U_{tr}+U_{T;W},\\ E_{T;S} & = & U_{tr}+U_{T;S},\\ E_{W;S} & = & U_{tr}+U_{W;S}. \end{array}$$ (31)

**Fig 8:**
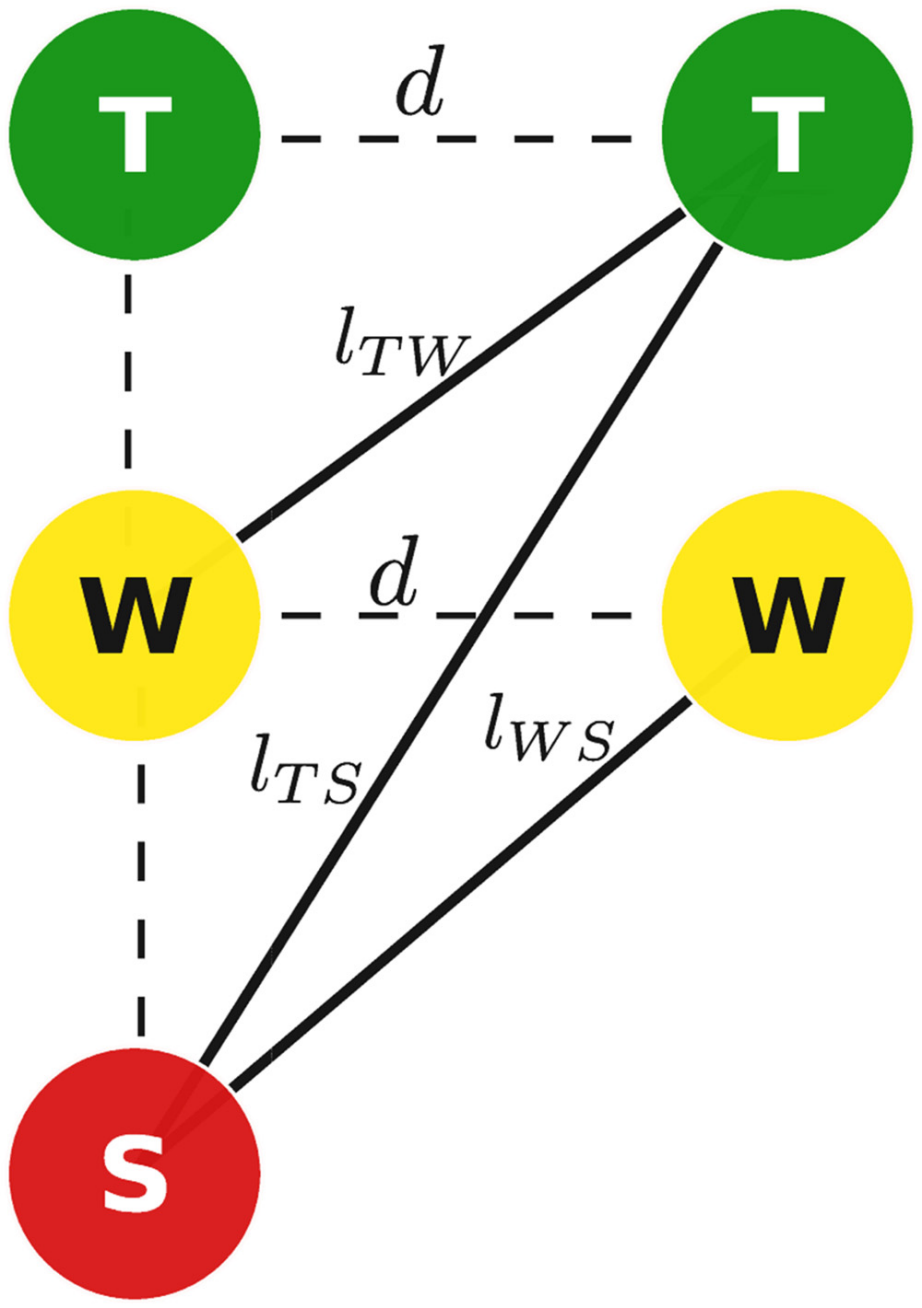
Scheme of possible conformations of tropomyosin between two cross-bridges. Here d is the length of one regulatory unit (36 nm), *l*_TW_, *l*_TS_ and *l*_WS_ are the lengths of tropomyosin at different conformations.

We assume that the lengthening of tropomyosin is related to tropomyosin displacement by

$$\begin{array}{c} d^{2}+<displacement{>}^{2}=<length{>}^{2}, \end{array}$$ (32)

and that the displacement for transition T → S is equal to the sum of displacements for transitions T → W and W → S, see Fig 8:

$$\begin{array}{c} \sqrt{l_{T;S}^{2}-d^{2}}=\sqrt{l_{T;W}^{2}-d^{2}}+\sqrt{l_{W;S}^{2}-d^{2}}. \end{array}$$ (33)

Combining Eqs (31) and (33), shows that the free energy component *U*_T;S_ can be calculated from

$$\begin{array}{c} U_{T;S}=U_{tr}{\left(\sqrt{1+{\left(\sqrt{\frac{U_{T;W}}{U_{tr}}+2\sqrt{\frac{U_{T;W}}{U_{tr}}}}+\sqrt{\frac{U_{W;S}}{U_{tr}}+2\sqrt{\frac{U_{W;S}}{U_{tr}}}}\right)}^{2}}-1\right)}^{2}. \end{array}$$ (34)

Since free energies *U*_T;W_ and *U*_W;S_ are significantly smaller than *U*_*tr*_, the following approximation can be used to find *U*_T;S_:

$$\begin{array}{c} U_{T;S}={\left(\sqrt[{4}]{U_{T;W}}+\sqrt[{4}]{U_{W;S}}\right)}^{4}. \end{array}$$ (35)

*Cross-bridge kinetics*. Cross-bridge formation in muscle fiber, that is the attachment and detachment of myosin head to actin binding site, is covered by Eq (27). The force generation was modulated by calcium, leading to the mix of the first and second order reactions in the model. Thus, Eq (27) was rewritten in general form as follows:

$$\begin{array}{c} \frac{\partial n_{A}\left(x,t\right)}{\partial t}\;+\;\frac{\partial n_{A}\left(x,t\right)}{\partial x}v\left(t\right)=\\ \;\sum_{B}(k_{B,A}\left(x,t\right)\;n_{B}\left(x,t\right)\;C_{B,A}\left(t\right)-k_{A,B}\left(x,t\right)n_{A}\left(x,t\right)\;C_{A,B}\left(t\right)), \end{array}$$ (36)

where *C*_*A*,*B*_(*t*) will be defined below. As described in general theory, only transitions involving a state change of only one cross-bridge in the group are allowed. Hence, the summation in Eq (36) involves only group indexes in the following form: *B* = (*α*_1_, …, *β*_*j*_, …, *α*_*q*_) where *A* = (*α*_1_, …, *α*_*j*_, …, *α*_*q*_).

Factor *C*_*A*,*B*_(*t*) depends only on *α*_*j*_ and *β*_*j*_ and can be written as

$$\begin{array}{c} C_{\alpha_{j},\beta_{j}}\left(t\right)=\{\begin{array}{cc} Ca(t) & \text{if}\;\;\left(\alpha_{j},\beta_{j}\right)\in \left\{(T,W_{\text{Ca}}),(S_{2},S_{2\text{Ca}})\right\},\\ 1 & \text{if}\;\;\left(\alpha_{j},\beta_{j}\right)\in \{(W_{\text{Ca}},S_{1\text{Ca}}),\\ & (S_{1\text{Ca}},S_{2\text{Ca}}),(W_{\text{Ca}},S_{2\text{Ca}}),(T,S_{2})\},\\ 0 & \text{otherwise.} \end{array} \end{array}$$ (37)

**Fig 9:**
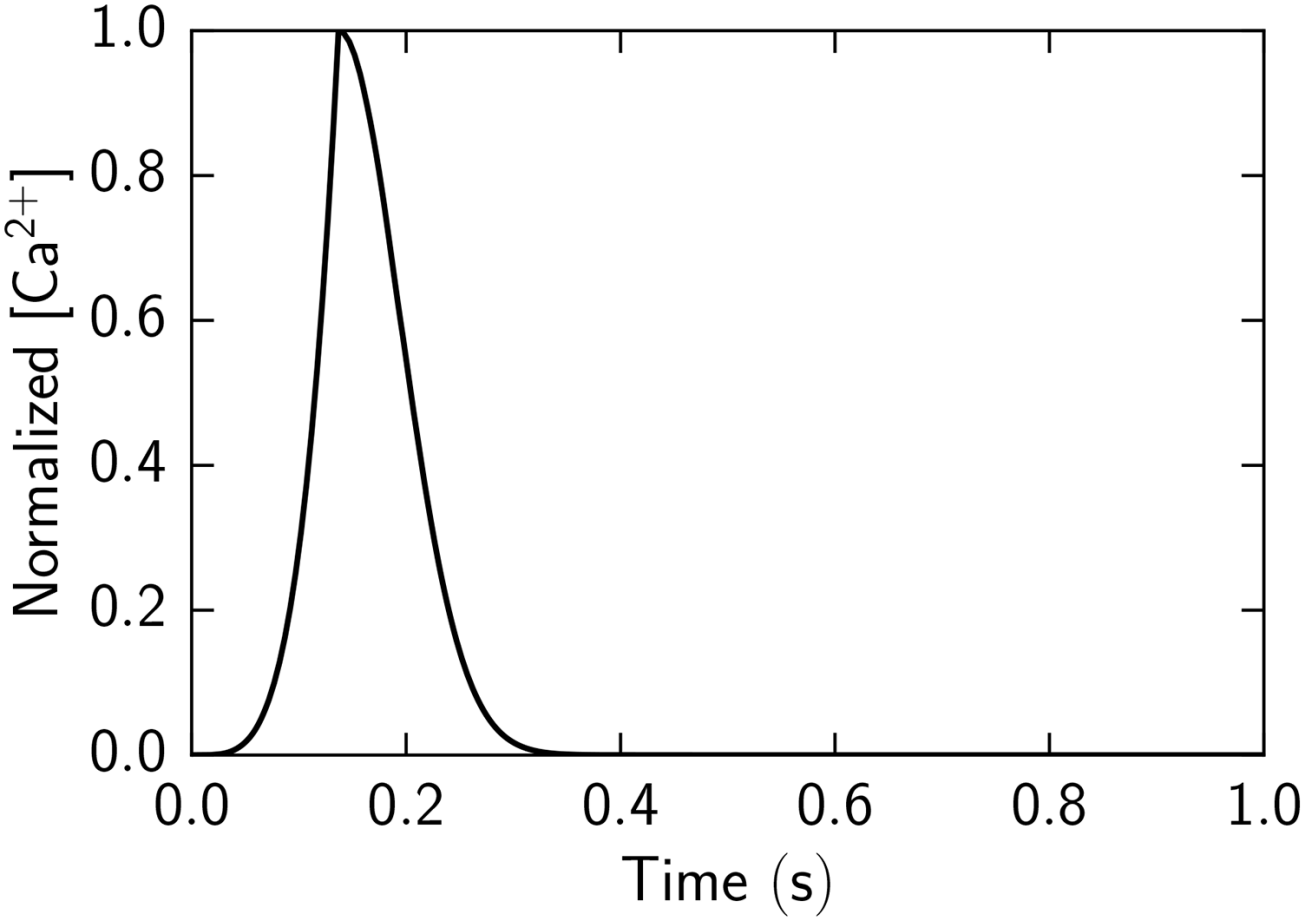
Normalized Ca^2+^ concentration transient used in simulations.

Here, *Ca*(*t*) describes Ca^2+^ transient as follows

$$\begin{array}{c} Ca\left(t\right)=\{\begin{array}{cc} {\left(\frac{t}{T_{p}}\right)}^{4} & \text{if}\;t<T_{p},\\ e^{-{\left(\frac{t-T_{p}}{T_{d}}\right)}^{2}} & \text{otherwise,} \end{array} \end{array}$$ (38)

where *T*_*p*_ is the time to peak of Ca^2+^, *T*_*d*_ is characteristic duration time as in [5]. In simulations model with cooperativity and without cooperativity, we used the same parameters to describe Ca^2+^ transient (Fig 9).

The rate constants were partitioned into components describing contribution of tropomyosin free energy change in reaction *h*_*A*,*B*_(*x*), dependence on cross-bridge position *f*_*α*_*j*_, *β*_*j*__(*x*) and sarcomere length *p*_*α*_*j*_, *β*_*j*__(*l*(*t*)), as follows:

$$\begin{array}{c} k_{A,B}\left(x,t\right)=e^{-\frac{G_{\beta_{j}}-G_{\alpha_{j}}}{2RT}}\;h_{A,B}\left(x\right)\;f_{\alpha_{j},\beta_{j}}\left(x\right)\;p_{\alpha_{j},\beta_{j}}\left(l\left(t\right)\right). \end{array}$$ (39)

Note that the first term in the product corresponds to the contribution of the free energy change of the cross-bridge undergoing the state change in the reaction.

The free energy difference of tropomyosin during transition from *A* to *B* and its contribution to rate constants was taken into acount through *h*_*A*,*B*_(*x*). Tropomyosin influence was incorporated into the rate constants by increasing the free energy of the product state of the forward reaction:

$$\begin{array}{c} h_{A,B}\left(x\right)=e^{-\frac{U_{\alpha_{j-1},\beta_{j}}+U_{\beta_{j},\alpha_{j+1}}-U_{\alpha_{j-1},\alpha_{j}}-U_{\alpha_{j},\alpha_{j+1}}}{RT}} \end{array}$$ (40)

if (*α*_*j*_, *β*_*j*_) ∈ {(W_Ca_, T), (S_1Ca_, W_Ca_), (S_2Ca_, S_1Ca_), (W_Ca_, S_2Ca_), (S_2Ca_, S_2_), (T, S_2_)}, otherwise *h*_*A*,*B*_(*x*) = 1.

The dependence of the rate constant on cross-bridge position and sarcomere length are symmetric:

$$\begin{array}{c} f_{\alpha_{j},\beta_{j}}\left(x\right)=f_{\beta_{j},\alpha_{j}}\left(x\right), \end{array}$$ (41)

$$\begin{array}{c} p_{\alpha_{j},\beta_{j}}\left(l\left(t\right)\right)=p_{\beta_{j},\alpha_{j}}\left(l\left(t\right)\right). \end{array}$$ (42)

The dependence of the rate constant on cross-bridge position *f*_*α*_*j*_, *β*_*j*__(*x*) was either constant (for reactions involving calcium binding) or continuous piecewise linear with given nodal points. The values of the nodal points were found by fitting. The location of the nodal points were as follows. For transitions between W_Ca_ and S_1Ca_, the nodal points were the boundary points *d*/2 and −*d*/2, S_1Ca_ free energy minimum, and the locations at which free energies of T and S_1Ca_ intersect. For transitions between S_1Ca_ and S_2Ca_, the nodal points were the boundary points *d*/2 and −*d*/2, S_2Ca_ free energy minimum (*x* = 0), the location at which S_1Ca_ and T free energies intersect after S_1Ca_ free energy minimum. For transitions between S_2Ca_ and W_Ca_, the same nodal points were used as for transitions between S_1Ca_ and S_2Ca_. For transitions between S_2Ca_ and T, the nodal points were the boundary points *d*/2 and −*d*/2, S_2Ca_ free energy minimum, the locations at which free energies of W_Ca_ and S_2Ca_ intersect, and the location found as a sum of S_1Ca_ free energy minimum location and the positive location at which S_2Ca_ and W_Ca_ free energies intersect.

Sarcomere length dependence *p*_*α*_*j*_, *β*_*j*__(*l*(*t*)) was introduced only for calcium binding reactions (transitions between T and W_Ca_, and between S_2_ and S_2Ca_) and myosin binding reaction to actin (transition between W_Ca_ and S_1Ca_). For all other transitions, *p*_*α*_*j*_, *β*_*j*__(*l*(*t*)) was taken equal to one. For transitions between T and W_Ca_ as well as between S_2_ and S_2Ca_, *p*_*α*_*j*_, *β*_*j*__(*l*(*t*)) was in the form

$$\begin{array}{c} p_{\alpha_{j},\beta_{j}}\left(l\right)=1+p_{\alpha_{j},\beta_{j}}^{L1}\;\frac{l_{max}-l}{l-l_{min}}, \end{array}$$ (43)

where $p_{\alpha_{j},\beta_{j}}^{L1}$ was optimized model parameter, *l*_*max*_ and *l*_*min*_ were 1.1 *μ*m and 0.8 *μ*m, respectively. For transition between W_Ca_ and S_1Ca_, we defined

$$\begin{array}{c} p_{W_{\text{Ca}},S_{1\text{Ca}}}\left(l\right)=\exp(p_{W_{\text{Ca}},S_{1\text{Ca}}}^{L2}\;{\left(\frac{l-l_{min}}{l_{max}-l_{min}}\right)}^{4})+1, \end{array}$$ (44)

where $p_{{\text{W}}_{\text{Ca}},{\text{S}}_{1\text{Ca}}}^{L2}$ was optimized model parameter.

*Total force and ATP consumption.* According to our assumption that only strong binding states produce force, the Cauchy stress *σ*_*a*_ developed by the cross-bridges in half-sarcomere is calculated according to the following equation:

$$\begin{array}{c} \sigma_{a}=\frac{ml}{2d}\sum_{A}\int_{-\frac{d}{2}}^{\frac{d}{2}}n_{A}F_{A}dx. \end{array}$$ (45)

The ATP consumption is given by the cross-bridge cycling rate:

$$\begin{array}{c} V_{ATP}=\frac{1}{d}\sum_{A}\int_{-\frac{d}{2}}^{\frac{d}{2}}\delta_{ATP}\left(A,B\right)(k_{B,A}n_{B}-k_{A,B}n_{A})dx, \end{array}$$ (46)

where

$$\begin{array}{c} \delta_{ATP}\left(B,A\right)=\delta_{ATP}\left(A,B\right)=\{\begin{array}{cc} 1 & \text{if}\;\text{index}\;j\;\text{exists}\;\text{such}\;\text{that}\\ & \left(\alpha_{j},\beta_{j}\right)\in \left\{\left(T,S_{2}\right),\left(W_{\text{Ca}},S_{2\text{Ca}}\right)\right\},\\ 0 & \text{otherwise}. \end{array} \end{array}$$ (47)

Then, the total ATP consumption during a cycle is:

$$\begin{array}{c} V_{ATP}^{beat}=\frac{1}{d}\sum_{A}\int_{0}^{T_{c}}\int_{-\frac{d}{2}}^{\frac{d}{2}}\delta_{ATP}\left(A,B\right)(k_{B,A}n_{B}-k_{A,B}n_{A})dx, \end{array}$$ (48)

where *T*_*c*_ is the period of a beat.

*Sarcomere dynamics*. In the mechanical protocols where the sarcomere is allowed to shorten or lengthen, the sarcomere lengthening rate *v*(*t*) is found by solving the equation

$$\begin{array}{c} \sigma_{a}\left(t\right)=<predefined\;stress\;state> \end{array}$$ (49)

for *v*(*t*) at each integration time step of Eq (27). For example, for physiological contraction, *v*(*t*) is found such that the force produced by the sarcomere is the same as an afterload during the shortening phase. In isotonic phase, *σ*_*a*_(*t*) = *const*. In isometric phase, the rate *v*(*t*) is set to zero.

### Fitting

*Residuals.* Model parameters were found by fitting the model solution to experimental data [20] with the goodness of the fit estimated by the least squares residuals. The least squares residuals were divided into three parts. The first part was obtained by comparing model solution to measured isometric force transients during a beat at different sarcomere lengths:

$$\begin{array}{c} R_{I}=\sum_{i=1}^{N_{isom}}{\left(\frac{P_{co}\left(l_{i},t_{i}\right)-P_{ex}\left(l_{i},t_{i}\right)}{\left(P_{max}-P_{co}\left(l_{i},t_{i}\right)\right)\sqrt{N_{isom}}/10}\right)}^{2}, \end{array}$$ (50)

where *P*_*co*_ and *P*_*ex*_ are computed and measured stress, respectively; *l*_*i*_ is half-sarcomere length; *t*_*i*_ is the time moment for measurement point *i*; *N*_*isom*_ is the number of measurement points.

The second part of residuals was obtained by comparing the end-systolic points of physiological contraction at different afterloads with the end-systolic points of isometric contraction. Here, the physiological contractions were calculated by the model, and end-systolic points of isometric contraction were taken from Janssen et al measurements [20]:

$$\begin{array}{c} R_{II}=\sum_{i=1}^{N_{isot}}{\left(\frac{l_{isom}^{es}\left(i\right)-l_{phy}^{es}\left(i\right)}{\left(l_{phy}-l_{min}\right)\sqrt{N_{isot}}/5}\right)}^{2}, \end{array}$$ (51)

where $l_{isom}^{es}$ and $l_{phy}^{es}$ are end-systolic half-sarcomere lengths for isometric and physiological contractions, respectively; *l*_*phy*_ is an end-diastolic half-sarcomere length for the physiological contraction (1.05 nm); *l*_*min*_ is the minimum length of half-sarcomere (0.8 nm); and *N*_*isot*_ is the number of different afterloads used in optimization.

The third part of residuals was obtained by comparing the total amount of consumed ATP molecules per myosin head during a cardiac cycle found by the model with the amount expected from SSA assuming 65% efficiency [5]. This comparison was done for isometrical and physiological contractions:

$$\begin{array}{c} R_{III}=\sum_{i=1}^{N_{SSA}}{\left(\frac{V^{beat}{\left(i\right)}_{ATP}/SSA\left(i\right)-\eta }{\eta \sqrt{N_{SSA}}}\right)}^{2}, \end{array}$$ (52)

where *V* ^*beat*^ and *SSA* are the total *ATP* consumption and stress-strain area, respectively; *η* is 0.142 kJ^−1^ m^3^ [5]; and *N*_*SSA*_ is the number of different afterloads and sarcomere lengths used in optimization.

*Optimization parameters.* In the optimization procedure, the model parameters are split into two sets. In the first set, the model parameter values were preset to certain values and all possible combinations of these parameter values were considered. For each of the combination of the model parameter values in the first set, the model parameter values in the second parameters set (see below) were optimized by the optimization algorithm. The optimal solution was found as one that had the smallest residual for all considered parameter sets.

The model parameters were divided into the two sets as follows. The first set consisted of five parameters describing free energy profiles shown at Fig 4A: $G_{{\text{S}}_{1}}^{min}$, *x*_1_ defines the value and location (relative to the S_2_ minimum) of minimal free energy in state S_1Ca_; $G_{{\text{S}}_{2}}^{min}$ is the minimal free energy in state S_2_; *U*_W;S_ and *U*_T;W_ are components determining the tropomyosin free energy. For simulations with *q* = 1, *U*_W;S_ and *U*_T;W_ were set to zero.

The second set consisted of parameters describing piecewise linear functions of rate constants of the cross-bridge transformation reactions (*f*_*α*,*β*_, Eq (41). Without the influence of tropomyosin and taking into account microscopic reversibility Eq (14), we have six independent cross-bridge cycling rates for every cross-bridge position. In addition, we assumed that Ca^2+^ association and dissociation rate constants are the same regardless to whether cross-bridge is in strong or weak binding state (transitions between W_Ca_ and T or S_2Ca_ and S_2_). As an example, rate profiles found after optimization are shown in Fig 4B.

Optimized parameters for model with *q* = 1 were used as an initial solution for model with *q* = 3.

### Numerical methods

The partial differential equations were discretized in cross-bridge position by the first order finite-difference method. The resulting system of ordinary differential equations was solved by using the DVODE package [32]. To speed up simulations, the original DVODE routines were modified to take into account the sparsity of the system and the parts of Jacobian matrix that were constant during a simulation.

For physiological contractions, the sarcomere lengthening rate *v*(*t*) was found by assuming that the rate is constant for each integration time interval. The rate was varied by hybrd solver from MINPACK package [33] until the calculated stress at the end of the time interval was the same as the given afterload. The procedure was repeated for the next time interval by taking the solution found for the end of the previous time interval as an initial condition. The time interval was taken initially to 1 ms and was reduced in the case of failure of nonlinear solver by 4 times.

The model parameters were found by minimizing the least squares residuals. The optimization was performed using the Levenberg-Marquardt algorithm [34] interfaced with the main program using F2PY [35]. Simulations were performed on the cluster of Linux/Intel Xeon E5-2630L computers.

## Supporting Information

S1 Text Appendix. (PDF)
