# Supplementary material for: Cross-Bridge Group Ensembles Describing Cooperativity in Thermodynamically Consistent Way
Source: PLoS One. 2015 Sep 11;10(9):e0137438. doi: 10.1371/journal.pone.0137438 (PMC4567334; doi:10.1371/journal.pone.0137438)
Supplement: S1 Text — (PDF) [file pone.0137438.s001.pdf]

Appendix:

Cross-Bridge Group Ensembles Describing Cooperativity in Thermodynamically Consistent Way

Mari Kalda, Pearu Peterson and Marko Vendelin
Laboratory of Systems Biology, Institute of Cybernetics, Tallinn University of Technology, Estonia

This appendix derives several equations describing cross-bridge dynamics. The appendix uses the same notations as the main text with only few new notations defined here.

Aim 1. Derivation of the equation describing dynamics of cross-bridge groups distribution function γ.

Derivation. The cross-bridge group cycling is described by dynamics of cross-bridge group density function $N_A(x_1,\ldots,x_q,t)$ with

$$\sum_A \int N_A(x_1,\ldots,x_q,t)dx_1\cdots dx_q = 1, \tag{A.1}$$

$$\begin{aligned} \frac{\partial N_A(x_1,\ldots,x_q,t)}{\partial t} &+ \frac{\partial N_A(x_1+\xi,\ldots,x_q+\xi,t)}{\partial \xi}\Big|_{\xi=0} v(t) \\ &= \sum_B (k_{B,A}N_B(x_1,\ldots,x_q,t) - k_{A,B}N_A(x_1,\ldots,x_q,t)), \end{aligned} \tag{A.2}$$

where $k_{A,B} = k_{A,B}(x_1,\ldots,x_q,t)$ are rate constants between biochemical states, $v(t)$ is the rate of the contractile element lengthening.
Let us define

$$N_A(x_1,\ldots,x_q,t) = \gamma(x_1,\ldots,x_q,t)n_A(x_1,\ldots,x_q,t), \tag{A.3}$$

$$\sum_A n_A(x_1,\ldots,x_q,t) = 1. \tag{A.4}$$

Using this notation, we can rewrite Eq. A.2 as

$$\begin{aligned} \frac{\partial\, \gamma(x_1,\ldots,x_q,t)n_A(x_1,\ldots,x_q,t)}{\partial\, t} + \frac{\partial\, \gamma(x_1+\xi,\ldots,x_q+\xi,t)n_A(x_1+\xi,\ldots,x_q+\xi,t)}{\partial\, \xi}\Big|_{\xi=0} v(t) &= \\ \sum_B (k_{B,A}\gamma(x_1,\ldots,x_q,t)n_B(x_1,\ldots,x_q,t) &- \\ k_{A,B}\gamma(x_1,\ldots,x_q,t)n_A(x_1,\ldots,x_q,t)) &. \end{aligned} \tag{A.5}$$

To find γ, we sum up all the equations over all $A \in \mathbb{S}^q$. Taking into account property of n_A (Eq. A.4) and that the sum of all reaction rates is zero (there are no sources or sinks for cross-bridges), dynamics of $\gamma(x_1,\ldots,x_q)$ is described by

$$\frac{\partial\, \gamma(x_1,\ldots,x_q,t)}{\partial\, t} + \frac{\partial\, \gamma(x_1+\xi,\ldots,x_q+\xi,t)}{\partial\, \xi}\Big|_{\xi=0} v(t) = 0. \tag{A.6}$$

The solution for Eq. A.6 with initial condition

$$\gamma(x_1,\ldots,x_q,t_0) = \gamma_0(x_1,\ldots,x_q) \tag{A.7}$$

is

$$\gamma(x_1,\ldots,x_q,t) = \gamma_0(x_1-a(t),\ldots,x_q-a(t)) \tag{A.8}$$

with

$$a(t) = \int_{t_0}^{t} v(\tau)d\tau. \tag{A.9}$$

Aim 2. Here we derive equations used to simulate dynamics of cross-bridge groups in the implemented model.

Derivation. In the implemented model, for the choice of γ_0 (in main text Eq. 26), γ is time independent and

$$\frac{\partial \gamma(x_1+\xi,\ldots,x_q+\xi,t)}{\partial \xi}\Big|_{\xi=0} = 0. \tag{A.10}$$

The Eq. A.5 simplifies to

$$\begin{aligned}\frac{\partial n_A(x_1,\ldots,x_q,t)}{\partial t} &+ \frac{\partial n_A(x_1+\xi,\ldots,x_q+\xi,t)}{\partial \xi}\Big|_{\xi=0} v(t) \\ &= \sum_B (k_{B,A} n_B(x_1,\ldots,x_q,t) - k_{A,B} n_A(x_1,\ldots,x_q,t)).\end{aligned} \tag{A.11}$$

The following coordinate transformation

$$x'_1 = \frac{1}{q}\sum_{i=0}^{q} x_i, \tag{A.12}$$

$$x'_2 = x_2 - x_1, \tag{A.13}$$

$$\ldots$$

$$x'_q = x_q - x_1, \tag{A.14}$$

turns Eq. A.11 in to $1+1$ dimensional PDE:

$$\frac{\partial n'_A}{\partial t} + \frac{\partial n'_A}{\partial x'_1} v(t) = \sum_B (k'_{B,A} n'_B - k'_{A,B} n'_A), \tag{A.15}$$

with n'_A and $k'_{A,B}$ representing n_A and $k_{A,B}$ in a new coordinate system $(x'_1,\ldots,x'_q)$.
